# Non-rheumatic streptococcal myocarditis mimicking acute ST-segment elevation myocardial infarction: characterization by cardiac magnetic resonance

**DOI:** 10.1186/1532-429X-11-S1-O69

**Published:** 2009-01-28

**Authors:** Rasoul Mokabberi, Jamshid Shirani, Afsaneh Haftbaradaran Mohammadi, William Schiavone

**Affiliations:** Geisinger Medical Center, Danville, PA USA

**Keywords:** Cardiac Magnetic Resonance, Late Gadolinium Enhancement, Invasive Coronary Angiography, Acute Myocarditis, Wall Motion Score Index

## Introduction

Acute myocarditis may mimic acute ST-segment elevation myocardial infarction (STEMI) and result in unnecessary invasive coronary angiography. Cardiac magnetic resonance (CMR) can differentiate myocarditis from acute ischemic myocardial injury and provide a basis for adoption of an appropriate treatment strategy in such patients.

## Purpose

The aim of this observation was to determine the diagnostic value of CMR in assessment of patients with acute non-rheumatic streptococcal myocarditis.

## Methods and results

We evaluated 6 young adults [age 22–35 (mean 29 ± 5) years, 5 men] with acute non-rheumatic streptococcal myocardits following recent (mean 4.8 days prior to presentation) pharyngitis (positive throat culture or rapid antigen test, elevated antistreptolysin antibody). All patients presented with non-pleuritic chest pain and focal ST segment elevation on admission electrocardiogram. Cardiac enzymes (creatine phosphokinase and troponin T) were elevated and emergent coronary angiography revealed normal coronary arteries. Two-dimensional transthoracic echocardiography showed regional wall motion abnormality (RWMA) with wall motion score index (WMSI) ranging from 1.12–1.24 and left ventricular ejection fraction (LVEF) ranging from 40–57%, and contrast-enhanced CMR demonstrated characteristic subepicardial late gadolinium enhancement (LGE) involving 4.8 ± 2.4 myocardial segments without an early perfusion defect (Table 1). Patients were treated with antibiotics and anti-inflammatory agents and showed clinical recovery within 3 days.

**Table 1:** Clinical and Imaging Characteristic in 6 Patients

Patient	1	2	3	4	5	6
Age (years)	27	35	33	32	29	22
Sex	M	M	M	M	F	M
ECG ST-Elevation	Inferolateral	Inferolateral	Lateral	Lateral	Inferolateral	Inferolateral
Echo RWMA	Inferolateral, lateral	Inferior, inferoseptal	Inferior, inferolateral	Inferior	Inferior, inferoseptal	Inferolateral
Echo WMSI	1.24	1.12	1.24	1.12	1.12	1.12
Echo LVEF (%)	40	45	45	57	52	51
Segments with LGE	NA	4	3	3	5	9

## Conclusion

Acute non-rheumatic streptococcal myocarditis may mimic STEMI. Clinical history (young individual, preceding pharyngeal infection), laboratory evidence of acute streptococcal infection and characteristic CMR findings can distinguish these patients from those with acute STEMI who benefit from primary coronary intervention.

